# Supplementary material for: Reaction of Partially Methylated Polygalacturonic Acid with Iron(III) Chloride and Characterization of a New Mixed Chloride–Polygalacturonate Complex
Source: Molecules. 2024 Feb 17;29(4):890. doi: 10.3390/molecules29040890 (PMC10893460; doi:10.3390/molecules29040890)
Supplement: Supplementary file 1 [file molecules-29-00890-s001.zip › molecules-2847031-supplementary.pdf]

Electronic Supplementary Information

Reaction of partially methylated polygalacturonic acid with iron(III) chloride and characterization of a new mixed chloride–polygalacturonate complex

László Kótai[1*] , Károly Lázár[2], László Ferenc Kiss[3], and Klára Szentmihályi[1]

[1]HUN-REN, Institute of Materials and Environmental Chemistry, Research Centre for Natural Sciences, H-1117 Budapest, Magyar tudósok körútja 2, Hungary

[2]HUN-REN, Centre for Energy Research, EKBI, Konkoly Thege Miklós út, 29-33, Budapest, 1121, Hungary

[3]HUN-REN Wigner Research Centre for Physics, Budapest, H-1525, Hungary

*Correspondence: kotai.laszlo@ttk.hu;

Table S1 The acid capacity of samples containing polygalacturonic acid.

Conditions of PGA preparation	Ion exchange temperature, °C	pH value of the ion-exchanged reaction mixture	Acid capacity of the samples, mekv/g	Relative acidity of the samples, %*
Commercial PGA	-	-	5.1	100
3 M NaOH, pH=12.2, 1 h, room temperature (Sample A)	25	2.84	3.6	71
		2.64	4.1	80
1 M NaOH, pH=12.2, 1 h, room temperature	25	2.86	2.9	57
		2.28	4.1	80
0.5 M NaOH, pH=12.1, 1 h, room temperature (Sample C)	25	3.31	2.6	51
		2.23	3.9	76
1 M NaOH, pH=12.0, 1 h, the reaction temperature was 50 °C (Sample D)	25	2.50	5.4	106
1 M NaOH, pH=12.0, 1 h, the reaction temperature was 50 °C (Sample E)	45-50	3.44	2.9	57
	45-50	2.55	3.7	73
	45-50	2.04	5.7	112
1 M NaOH, pH=12.0, 1 h, the reaction temperature was 75 °C (Sample F)	70-75	3.50	2.9	57
	70-75	2.55	4.1	80
	70-75	1.88	5.9	116

Table S2 Composition of compound **2.**

Overall Fe content	9.95 %
Fe^{III} content	9.62 %
Fe^{II} content	0.33 %
Carbon content	29.4 %
Chloride ion content	4.7 %
PG ring/Fe ratio from chloride titration	2.12
PG ring/Fe ratio from CHN analysis	2.11
Carboxylic acid/methylated carboxylate ratio in PGA	1:1

Table S3 Mössbauer parameters of compound **2*** measured at room temperature.

Compound	Component	IS, mm/s	QS, mm/s	FWHM, mm/s	RI
**PGA-Fe^{III}***	Fe^{III}(a)	0.38	0.62	0.30	34
	Fe^{III}(b)	0.39	1.10	0.44	48
	Fe^{II}(a)	0.97	2.16	0.53	7
	Fe^{II}(b)	1.34	2.28	0.52	12

Table S4 XPS parameters of compound **2** and basic zinc and magnesium polygalacturonates

Compound	Energy, eV	
Compound **2**	284.899	C_{1s}
	286.680	
	288.568	
Mg(OH)PG	284.642	
	286.060	
	287.812	
Zn(OH)PG	284.933	
	286.814	
	288.815	
Compound **2**	532.395	O_{1s}
Mg(OH)PG	531.759	
	533.140	
Zn(OH)PG	532.919	
Compound **2**	710.365	$Fe_{2p3/2}$
	722.942	$Fe_{2p1/2}$
	713.548	$Fe_{2p3/2}$
	724.707	$Fe_{2p1/2}$
Mg(OH)PG	50.709	Mg_{2p}
Zn(OH)PG	1022.698	$Zn_{2p3/2}$
	1045.757	$Zn_{2p1/2}$

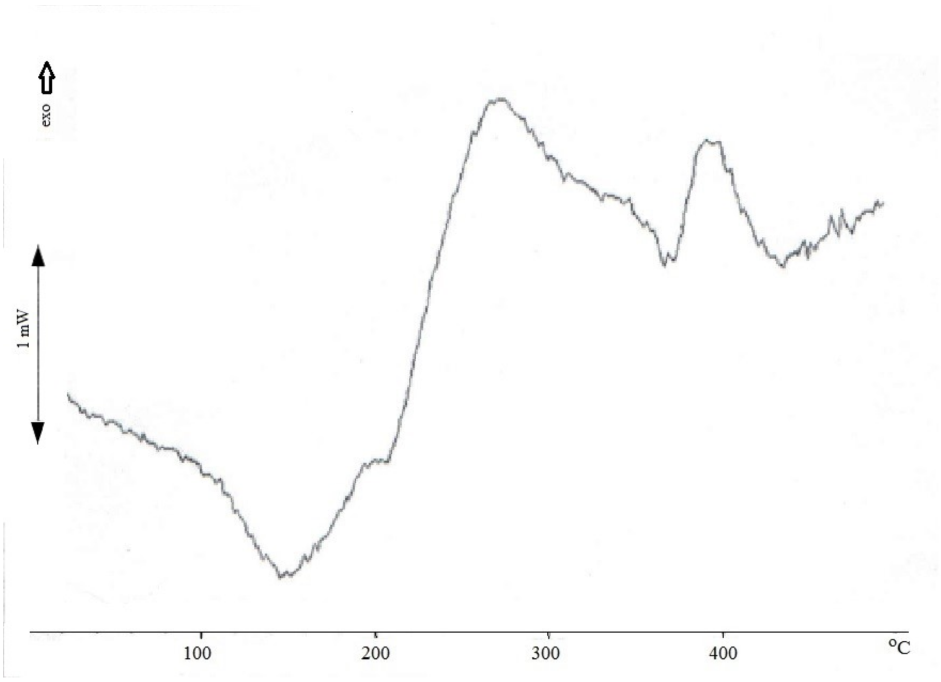

Figure S1 DSC curve of compound **2** under N_2

a)

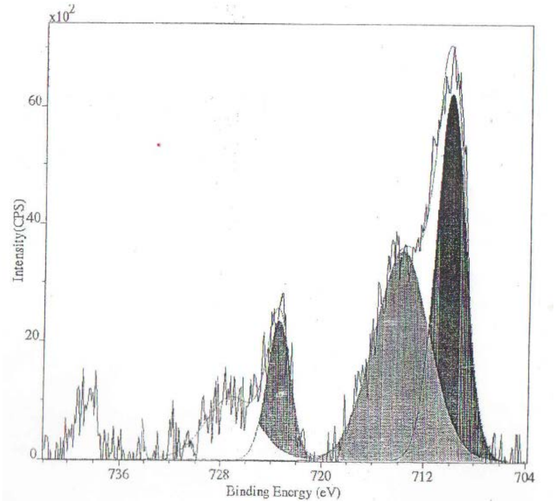

b)

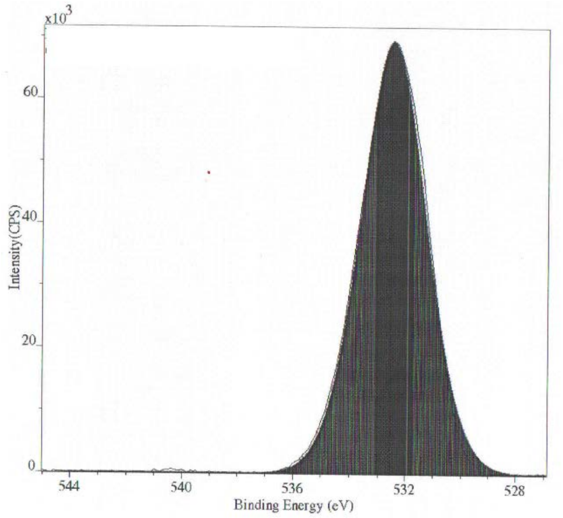

c)

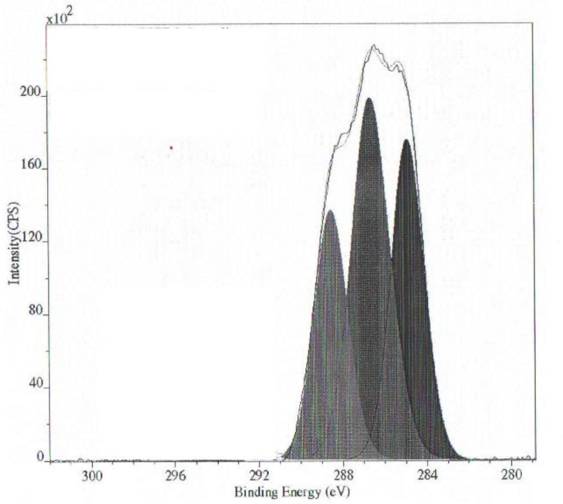

Figure S2 Fe 2p (a), O1s(b) and C1s (c) XPS spectra of compound 2.

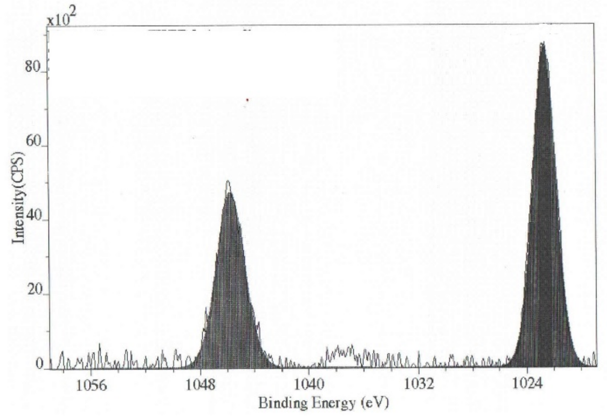

Figure S3 Zn 2p XPS spectrum of basic Zn polygalacturonate.

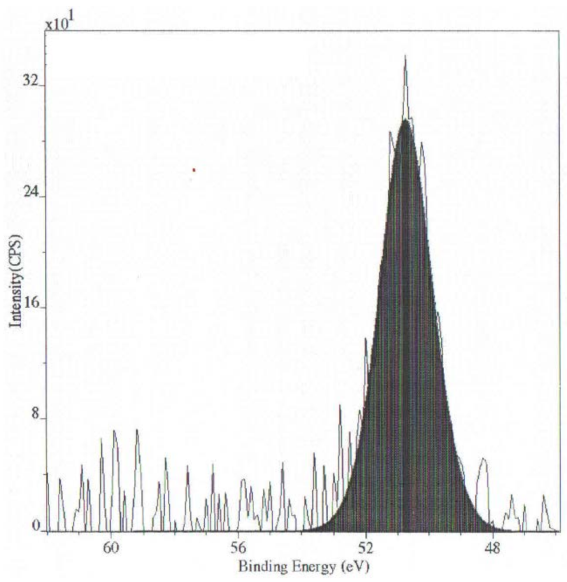

Figure S4 Mg 2p XPS spectrum of basic Mg polygalacturonate.

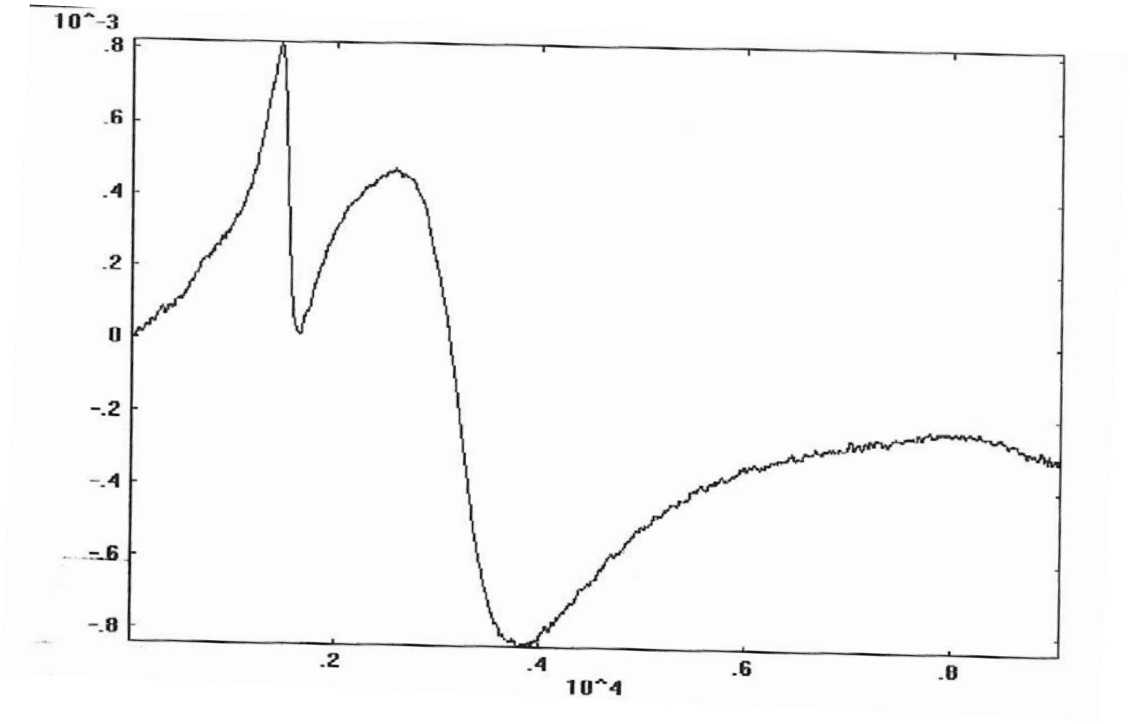

Figure S5 ESR spectrum of compound **2**.

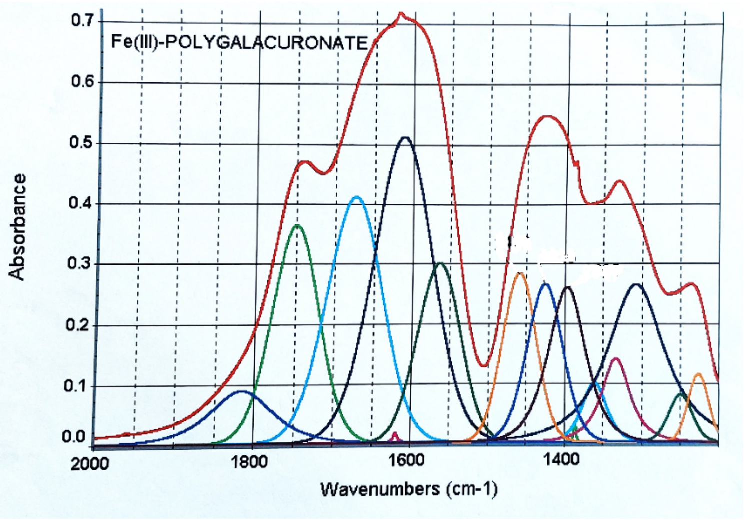

Figure S6 IR spectrum of compound **2** in the carboxylate region (different colors used to distinguish each spectral band component).

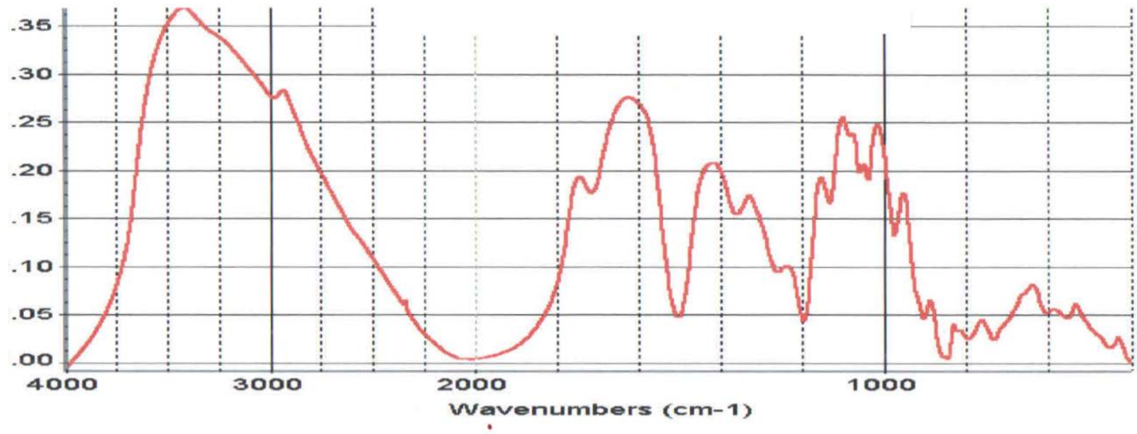

Figure S7 IR spectrum of compound 2 between 4000 and 400 cm^{-1}.

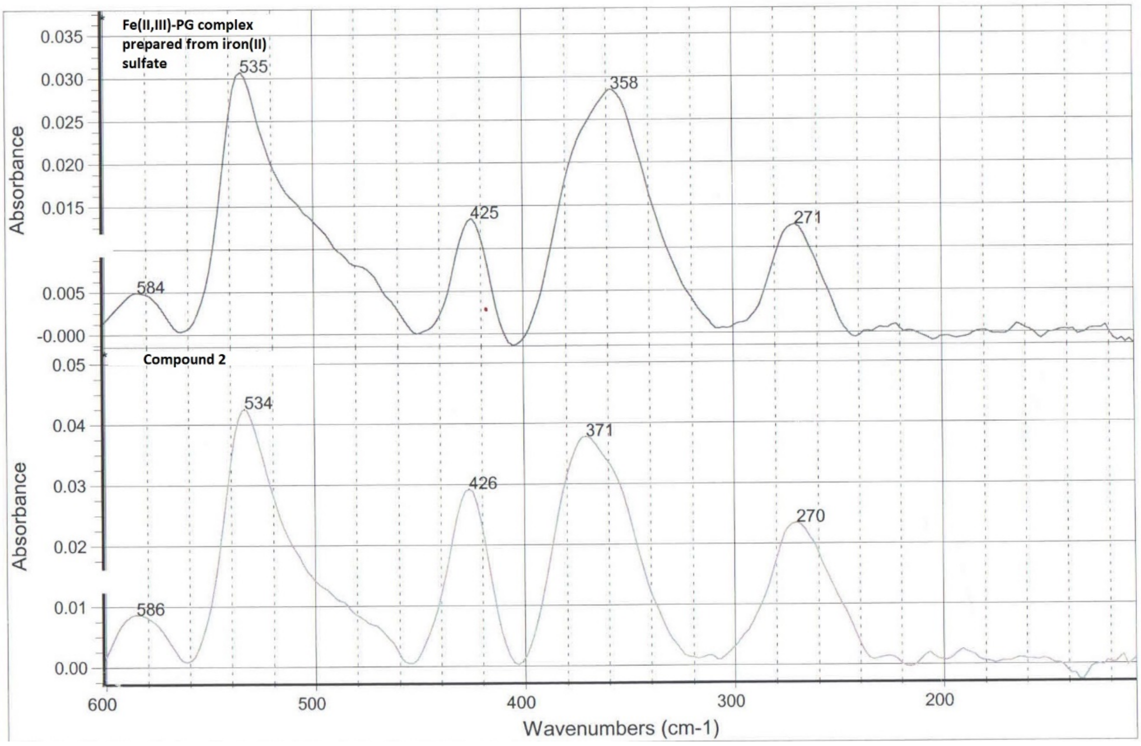

Figure S8 Far-IR spectra of a Fe(II,III) mixed valence polygalacturonate complex prepared from iron(II) sulfate and compound **1**.
